# Supplementary material for: Comorbidities affecting re-admission and survival in COVID-19: Application of joint frailty model
Source: PLoS One. 2024 Apr 18;19(4):e0301209. doi: 10.1371/journal.pone.0301209 (PMC11025956; doi:10.1371/journal.pone.0301209)
Supplement: Attachment — Submitted filename: manuscript_Reviewed.docx [file pone.0301209.s003.docx]

**Comorbidities affecting re-admission and survival in COVID-19: Application of joint frailty model**

**Short title:** Comorbidities affecting re-admission and survival in COVID-19

Pouyan Ebrahimi^1a^, Akram Yazdani^2a^, Seyyed Ali Mozaffarpur^3a^, Hamed Mehdinejad^4,5^, Hoda Shirafkan^6^*

1- Student Research Committee, Babol University of Medical Sciences, Babol, Iran
2- Department of Biostatistics and Epidemiology, Faculty of Health, Kashan University of Medical Sciences, Kashan, Iran
3- Traditional Medicine and History of Medical Sciences Research Center, Health Research Institute, Babol University of Medical Sciences, Babol, Iran.
4- Infectious Diseases and Tropical Medicine Research Center, Health Research Institute, Babol University of Medical Sciences, Babol, Iran.
5- Clinical Research Development Unit of Rouhani Hospital, Babol University of Medical Sciences, Babol,Iran.
6- Social Determinants of Health Research Center, Health Research Institute, Babol University of Medical Sciences, Babol, Iran.

*Corresponding author:

email: [hodashirafkan@gmail.com](mailto:hodashirafkan@gmail.com) (HSh)

<https://orcid.org/0000-0002-5682-1153>

^a^ SAM, PE and AY contributed equally to this work.

**Abstract:**

**Background****:**

A common concern in healthcare systems caused by COVID-19 is the potential re-admission for patient. In addition to adding costs to the healthcare system, re-admissions also endanger patient safety. Recognizing the factors that influence re-admission, can help provide appropriate and optimal health care. The aim of this study was to assess comorbidities that affect re-admission and survival in COVID-19 patients using a joint frailty model.

**Methods:**

This historical cohort study was done using data of patients with COVID-19 who were re-hospitalized more than 2 times in a referral hospital in North of Iran. We used the joint frailty model to investigate prognostic factors of survival and recurrence, simultaneously using R version 3.5.1 (library “frailtypack”). P-values less than 0.05 were considered as statistically significant.

**Results:**

A total of 112 patients with mean age of 63.76 (±14.58) years old were recruited into the study. Forty-eight (42.9%) patients died in which 53.83% them were readmitted for a second time.. Using adjusted joint model, the hazard of readmission increased with cancer (Hazard ratio (HR)=1.92, Standard error (SE)=0.16) and hyperlipidemia (HR=1.22, SE=0.22). Furthermore, The hazard of death increased with cancer (HR=1.64, SE=0.43) and hyperlipidemia (HR=4.05, SE=0.66) and decreased with lung disease (HR=0.11, SE=0.92), hypothyroidism (HR=0.32, SE=0.82) and hypertension (HR=0.97, SE=0.41). The hazard of death increased with increasing age (HR=1.76, SE=0.24).

**Conclusion:**

Considering the correlation between re-admission and mortality in the joint frailty model, malignancy and hyperlipidemia increased the risk of both re-admission and mortality. Moreover, lung disease was a protective factor against both mortality and re-admission.

**Keywords: Recurrent event analysis; Joint models; SARS-COVID-2; Survival; COVID19**

**Introduction**

COVID-19 has caused problems in healthcare systems. A common concern is the possibility of patients getting readmitted to hospital. Re-admission, in addition to imposing additional burden on the health care system, also endangers the safety of patients. By recognizing the factors affecting readmission, management and policy planning can be helpful in providing appropriate and optimal health care (1). As COVID-19 can have long-lasting implications on the health of individuals, more attention is paid to people who are at a higher risk of developing complications, such as the elderly and people with comorbidities like diabetes mellitus (DM) and hypertension (HTN). . Individuals with underlying comorbidities have been found to have poor prognosis.(2). For instance, DM patients were found to have a higher risk of morbidity and mortality, which can lead to hospitalizations and intensive care unit )ICU( admissions (3).

In some diseases, multiple events may occur after initial treatment due to the physical progression of the disease. For example, breast cancer patients may experience breast cancer recurrence, metastasis, or death after initial treatment (4). In COVID-19 infection, patients after initial treatment and discharge, may be re-hospitalized with different causes (including respiratory distress, metabolic encephalopathy, etc.) (1). Thus, to analyze recurrent events, we propose a shared frailty model taking into account the time to readmission or death to correlate different events among participants (5).

The conventional frailty model assumes that all patients experience the event of interest with varying non-zero risks (6). It is an extension of the Cox model to control heterogeneity between individuals (7). Observations of repeated events in longitudinal studies can be terminated by several types of events, such as study termination, loss to follow-up, or major failure events such as death. In some cases, major failure events may be correlated with the recurrence of major events such as shortness of breath. Therefore, it may violate the assumption of non-informational censorship (8). This dependency is incorporated into the joint modeling of deaths and recurring events. In this model, the effects of frailty on recurrent events and mortality are not the same, and estimates are made using the Monte Carlo EM algorithm (9).

In this study, we assess the comorbidities that affect readmission and survival in COVID-19 patients, using penalized probabilities of hazard functions to assess relapse and terminal event processes. We consider the correlation between readmission and mortality risk through the general gamma-frailty model. Understanding the factors that influence the risk of death in readmitted patients may help policymakers optimize care.

**Materials and Methods**

**Study design:**

This retrospective cohort study was conducted with patients with COVID-19 who were readmitted after the initial admission and discharge at Ruhani hospital in Babol, Iran from February 2020 to July 2021 at. Ruhani hospital is the biggest general hospital in North of Iran that covers more than 5 million residents in two Northern provinces of Iran.

All patients with multiple (at least two) hospitalizations for Covid-19 since the beginning of the Covid pandemic in Iran (February 2020) were included in the study. All of these patients had follow-ups during their hospital stay and until the end of the study (July 2021). For some of these patients, he was hospitalized more than once during the study period and that information was also collected. In July 2021, we called the patient who was discharged alive during his last hospitalization and obtained the patient's last status (mortality) on this call.

The recruitment process and collection of study data were conducted by investigators (A pulmonologist and A medical student). Written informed consent was obtained at each admission and verbal informed consent was obtained at the telephone call.

Ethics approval for this study was obtained from the Ethics Committee of the Babol University of Medical Sciences (IR.MUBABOL.REC.1400.187(.The researchers followed the principles of the Helsinki Declaration.

**Participants:**

Patients who met the following eligibility criteria were recruited into the study.

**Inclusion criteria:**

Patients with age more than 17 years old, confirmation of COVID-19 (positive reverse-transcriptase–polymerase-chain-reaction (RT-PCR)), or having COVID pneumonia (confirmed by imaging or laboratory documents), and readmission due to COVID-19 were recruited for the study.

**Exclusion criteria:**

Patients whose records were not complete; pregnant patients; and patients that discharging from the hospital with personal consent were excluded from the study.

**Patient discharge criteria:**

The discharge criteria was uniform for all patients according to the WHO clinical guidance for COVID-19 (11) (including no fever for 3 consecutive days, improving in respiratory symptoms, and evident resolution and improvement of the acute lung lesion in imaging)

**Study variables:**

Gender, age, time and duration of hospitalization, mortality, history of underlying comorbidities (including DM, HTN, cancer, heart disease, lung disease, kidney disease, and hyperlipidemia (HLP)), respiratory rate, oxygen saturation, and having any supplemental oxygen therapy were recorded. Assessing the severity of the disease, we used national early warning score for COVID-19 (NEWS2) (12). NEWS2 has 8 questions and each queation is scored between 0 to 3. The NEWS2 is categorized as low for scores 0 to 4, medium for scores 5 to 6 and more than 7 as high and score of 3 in any individual parameter as low-medium. Evaluating the current status of mortality, we followed all patients after the last dischargeby a telephone call in June and July 2021.

**Statistical analysis:**

Descriptive statistics were presented as mean (±standard deviation-SD), mean difference (MD), median, mode, interquartile range (IQR), and frequency (percent) for quantitative and qualitative data, respectively. We assessed the normality of variables by the Kolmogorov-Smirnov test. To evaluate the characteristics and comorbidities between survivors and dead patients we used independent t-test and chi-square test. We used the joint frailty model to investigate prognostic factors of survival and recurrence, simultaneously.

**Joint frailty model:**

The joint frailty model models the death and recurrent event processes simultaneously by a shared random effect. In summary, the hazard functions for the recurrent event and the death processes at time t can be expressed as

(1)

$$r_{ij}\left( t | v_{i} \right)=r_{0}\left( t \right)\exp\left( \beta\boldsymbol{Z}_{i}+v_{i} \right)$$

$$\lambda_{i}\left( t | v_{i} \right)=\lambda_{0}\left( t \right)exp(\alpha\boldsymbol{Z}_{i}+gv_{i})$$

in which i shows patient and j the recurrent event, we show the vector of covariate for each patient as Z_i_, and $\alpha, \beta$ as the regression coefficient for the recurrent event and death, respectively. In this model, g estimates the correlation between death and recurrent event processes. The baseline (underlying) hazard function for recurrent event and death are represented as $r_{0}\left( t \right)$ and$\lambda_{0}\left( t \right)$, respectively. Furthermore, we show the shared random effect as$v_{i}$. We assume the distribution of $v_{i}$ is normal with mean 0 and variance of j. This variance shows the unobservable heterogeneity between patients.

The statistical analysis was done using R version 3.5.1 (function " frailtyPenal" in the library “frailtypack”) and STATA version 14.2. P-values less than 0.05 were considered as statistically significant.

**Results**

A total of 112 patient with the mean (±SD) age of 63.76 (±14.58) (median: 65 IQR:51 to 72; 75 (67%) more than 60 years old) were recruited into the study; from which 48 (42.9%) patients died. Fifty-three and eighty-three percent (53.83%) of mortalities were at the second readmission with an average of 76 (±108) days (median 30, IQR 266, mode=13 days) after the first hospitalization. Also, 53 (47.32%) patients were re-hospitalized at least 3 times and 30 patients received ICU care at least once. Sixty nine (61.6%) patients were male. The median length of hospital stay was 6 days (IQR: 7: 3 to 10, mean ±SD: 8.33±8.63). Patients have been hospitalized with a total of 363 patient-time and an average of 2.70 (±2.01) times (Table 1).

**Table 1**: **Frequency of readmissions, survival and death among patients**

|  | **Readmission**  **(n=112)** | **Survived**  **(n=74)** | **Dead**  **(n=48)** |
| --- | --- | --- | --- |
| **First hospitalization** | - | 112 | 0 |
| **Second hospitalization** | 112 | 89 (79.5%)* | 23 (20.5%) |
| **Third hospitalization** | 53 | 46 (86.8%) | 7 (13.2%) |
| **Fourth hospitalization** | 31 | 24 (77.4%) | 7 (22.6%) |
| **Fifth hospitalization** | 18 | 16 (78.6%) | 2 (21.4%) |
| **Sixth hospitalization** | 14 | 11 (78.6%) | 3 (21.4%) |
| **Seventh hospitalization** | 10 | 8 (80.0%) | 2 (20.0%) |
| **More than eight hospitalization** | 4 | 0 | 4 (100%) |

*The percentages are within each hospitalization (row percentages)

Comparing the baseline characteristics of the patients, no differences were observed between age and gender of patients between groups (group A: patients who were discharged and group B: patients who died). Assessing underling diseases between two groups, we saw that the mortality rate among patients with cancer was higher than patients without cancer (OR=2.79, p-value=0.04), (Table 2).

**Table 2:** **Baseline characteristics and their comparisons between surviving status of COVID-19 patients with readmissions**

|  | **Total***  **(n=112)** | **Died**  **(n=48)** | **Discharged**  **(n=64)** | **p-value** |
| --- | --- | --- | --- | --- |
| **Sex (Male)** | 69 (61.6%) | 29 (60.4%) | 43 (62.5%) | 0.822 |
| **Age (year)** | 63.77(±14.58) | 65.48(±14.16) | 62.58(±14.72) | 0.297 |
| **HLP^a^** | 19 (17.00%) | 11 (23.4%) | 8 (12.5%) | 0.132 |
| **Kidney diseases** | 13 (11.6%) | 8 (17.0%) | 5 (7.8%) | 0.135 |
| **Lung disease** | 13 (11.6%) | 3 (4.7%) | 10 (15.7%) | 0.135 |
| **Heart disease** | 48 (42.9%) | 21 (44.7%) | 27 (42.2%) | 0.793 |
| **Cancer** | 19 (17.0%) | 12 (25.5%) | 7 (10 .9%) | **0.044** |
| **Hypothyroidism** | 9 (8.0%) | 3 (6.4%) | 6 (9.4%) | 0.568 |
| **DM^b^** | 52 (46.4%) | 24 (50.0%) | 28 (43.8%) | 0.445 |
| **HTN^c^** | 49 (43.8%) | 21 (44.7%) | 28 (43.8%) | 0.922 |

* All percentages are column percent and significant p-values are shown as bold text.

^a^HLP: Hyperlipidemia

^b^DM: Diabetes mellitus

^c^HTN: Hypertension

Assessing the severity of COVID-19 by NEWS2, we found that majority of patients were at high severity (n=39, 36.8%) in the first hospitalization (the severity of COVID-19 were at low, low to medium, and medium were 24 (22.6%), 18 (17.0%), and 25 (23.6%) patients, respectively). Evaluating the mortality of patients, we found that 53.8% of patients with high severity died.

**Primary symptoms:**

The most common COVID-19 symptoms among 363 patient-times were shortness of breath in 154 (42.42%), fever in 92 (25.34%), cough in 77 (21.21%) and fatigue in 108 (29.75%).The other symptoms were sore throat (n=2, 0.5%), chills (n=72,19.83%), headache (n=24, 6.61%), diarrhea (n=9, 2.47%), chest pain (n=21, 5.78%), sweating (n=11, 3.30%), abdominal pain (n=27, 7.44%), myalgia (n=23, 6.34%), loss of appetite (n=51, 14.05%), nausea and vomiting (n=56, 15.43%), and impaired consciousness (n=22, 6.06%).

The number of re-admissions varied from 1 to 13, with the average of 2.70 and median of 2 times per patient. We categorized episodes into the number of observation times for each patient. The last episode corresponds to a death or censoring time (discharge or after the last follow-up through the phone call).

**Prognostig factors for re-admission and mortality:**

Table 3 presents the results using adjusted joint model. The hazard of recurrence of readmission increased with cancer (HR=1.92, Standard error [SE]=0.16) and with HLP (HR=1.22, SE=0.22).

**Table 3:** **Factors affecting recurrent of hospitalization and survival of COVID-19 patients using joint frailty model**

|  | **recurrent event**  **(re-hospitalization)** | | **terminal event**  **(Death)** | |
| --- | --- | --- | --- | --- |
| **Variables** | **HR (SE)** | **p-value** | **HR (SE)** | **p-value** |
| **gender (male)** | 0.79 (0.15) | 0.144 | 0.67 (0.36) | 0.290 |
| **age** | 0.90 (0.08) | 0.207 | 1.76 (0.24) | 0.022 |
| **HLP** | 1.22 (0.22) | 0.363 | 4.05 (0.66) | 0.035 |
| **Kidney diseases** | 0.81 (0.24) | 0.397 | 1.74 (0.62) | 0.369 |
| **lung disease** | 0.61 (0.27) | 0.074 | 0.11 (0.92) | 0.019 |
| **heart** | 0.92 (0.15) | 0.603 | 0.88 (0.38) | 0.754 |
| **cancer** | 1.92 (0.16) | <0.001 | 1.64 (0.43) | 0.254 |
| **hypothyroidism** | 0.96 (0.26) | 0.896 | 0.32 (0.82) | 0.181 |
| **DM** | 0.89 (0.16) | 0.499 | 4.77 (0.49) | 0.001 |
| **HTN** | 0.98 (0.15) | 0.933 | 0.97 (0.41) | 0.943 |

The hazard of death increased with cancer (HR=1.64, SE=0.43), DM (HR=4.77, SE=0.49), and HLP (HR=4.05, SE=0.66). Also, it decreased with lung disease (HR=0.11, SE=0.92), hypothyroidism (HR=0.32, SE= 0.82), and HTN (HR=0.97, SE=0.41). Furthermore, the hazard of death increased with increasing age (HR=1.76, SE=0.24). The baseline survival functions for death and recurrent events (joint frailty model) was shown in Fig 1. As seen, survival declines more steeply over time as the number of readmissions increases.

**Fig 1**: **Baseline survival functions for mortality and recurrent events** (joint frailty model).

**Discussion**

In this study, we used a joint frailty model to find prognostic factors for the survival of COVID-19 patients considering the correlation with the recurrence of their re-hospitalization. Based on our best knowledge, the joint study of readmission and survival among COVID-19 patients has not been done, yet. Using this model, we found that malignancy and hyperlipidemia were risk factors for both the occurrence of mortality and also readmission. Moreover, chronic lung disease was a protective factor against mortality and recurrent hospitalization.We found 112 confirmed cases, which needed readmission. We followed up all patients and determined the survival status of them. We did not see any differences in age and gender between patients who were discharged and died . In a meta-analysis, [ClaraBonanad](https://www.sciencedirect.com/science/article/pii/S1525861020304412#!) et al (2020) emphasized the effect of age on mortality with the thresholds of age >50 years and, particularly, >60 years (13). The effect of age on mortality has also been shown in other studies(14), too. In our study, the hazard of mortality increased with age.

An important affective factor of patient 30-days all-cause readmissions is comorbidity with other diseases. So this factor has been utilized in risk assessment models (15). In our study, most patients had at least one chronic condition, consistent with other studies (1).

In our study cancer increased the hazard of both recurrent admissionsand mortality. It was seen that among readmitted patients, malignancy history was more prevalent. Especially, those patients who were readmitted to thrombotic episodes had a history of Malignancy, emphasizing the contribution of malignancy to the already existing hypercoagulability risk in COVID-19 (16). L.Zhang et al (2020) observed that cancer patients display deteriorating situations and negative consequences from the COVID-19 infection (17). [Ludi Yang](https://pubmed.ncbi.nlm.nih.gov/?term=Yang%20L%5BAuthor%5D) et al (2020) in a systematic review study examined the consequences of cancer in COVID-19 patients. They showed that cancer patients were more susceptible to COVID-19. As a risk factor, cancer increased mortality in COVID-19 patients. Among cancer patients with COVID-19, those with lung cancer had a higher mortality rate than those without lung cancer (18).

Chronic lung disease was more common in patients who required readmission than in those who did not, highlighting its potential impact on the development of dyspnea. It is a common cause of COPD exacerbations and increases the risk of subsequent readmission(19). [JaimeSignes-Costa](https://www.sciencedirect.com/science/article/pii/S0300289620305354#!) et al. (2021) showed that previous lung disease was a risk factor for mortality in patients with COVID-19 (20). [Jacobo Rogado](https://www.sciencedirect.com/science/article/pii/S0169500220304682#!) et al. (2020) showed that covid-19 patients who already had lung cancer have a higher mortality rate than the general population (21). In our study lung disease did not have significant effect on re-hospitalization but the effect size was considerable. Furethermre, it decreased the hazard of mortality which could be due to the use of corticosteroid due to the lung disease before contracting COVID-19.

Additionally, some cardiovascular disease patients had to be readmitted for drug-related adverse events. Specifically, antihypertensive regimens combined with diarrhea due to COVID-19 resulted in episodes of electrolyte imbalance and hypotension, which manifested as altered mental status at presentation. In our study hypothyroidism disease and high hypertension decreased the hazard of mortality and did not have any significant assertion with re-hospitalization. So-Young Kim examined the association between susceptibility, morbidity and mortality due to COVID-19 and hypothyroidism disease and found that a history of hypothyroidism disease was not associated with an increased risk of morbidity and mortality of COVID-19 (22). Furthermore, Yanbin Du et al. (2020) in their meta-analysis proposed that hypertension was independently associated with a significantly increased risk of severe COVID-19 and also in-hospital COVID-19. (23)

**Advantages of the study**

A key advantage of the joint frailty model is the ability to analyze recurring events and associated terminating event data simultaneously. In addition, this model can also evaluate the dependencies (8). Elimination of important aspects of dependency in the data could result in inefficient and biased estimates (8). Using frailty models not only introduce a comprehensive idea of the potential dependencies but also refines the probable bias for regression coefficient in reduced models like Cox proportional hazards models or recurrent event models (24, 25).

Another advantage of this model is that it allows for informative censoring, leading to more accurate estimations(26)

To evaluate the latest patient mortality status, we followed up all readmitted patients after the last hospitalization. Another advantage of our study is its design that allow us to assess the prognostic factors for readmission and survival.

**Limitations of the study**

We entered into the study all patients that were suspected of COVID-19 or whose RT-PCR was positive. As a limitation of our study, we did not assess the lung and chest imaging findings of the readmitted patients, this was beyond the scope of our research. Another limitation of our study, we consider all readmitted patients at a hospital in Babol in the north of Iran. This is a referral COVID-19 hospital, and patients with worse conditions refer to this hospital. This might affect the generalizability of the study results. As a suggestion for more studies, readmitting patients with narrower eligibility criteria could have more consistent results.

**Conclusion**

In our cohort study, about half of the patients were readmitted at least 3 times and the first readmission for them was within the first month after the first discharge. This is probably tended to be associated with the index medical institution course and eventually to COVID-19, highlighting that some readmissions were due to latent errors or omissions in hospital care. Considering the correlation with the re-hospitalization and mortality in a joint frailty model, malignancy and HLP increased the hazard of both re-hospitalization and mortality. Furthermore, having lung diseases was a protective factor for both mortality and re-hospitalization. Therefore, advanced statistical models can be used to more accurately identify risk factors for readmission and mortality, which can be used in continuing care to prevent patient readmission and death.

**Acknowledgments:**

The authors of this article express their appreciation and gratitude to the student research committee of Babol university of medical sciences and the clinical research development center of rouhani hospital.

**References**

1.	Atalla E, Kalligeros M, Giampaolo G, Mylona EK, Shehadeh F, Mylonakis E. Readmissions among patients with COVID‐19. International journal of clinical practice. 2021;75(3):e13700.

2.	Gupta R, Ghosh A, Singh AK, Misra A. Clinical considerations for patients with diabetes in times of COVID-19 epidemic. Diabetes & metabolic syndrome. 2020;14(3):211.

3.	Singh AK, Gupta R, Ghosh A, Misra A. Diabetes in COVID-19: Prevalence, pathophysiology, prognosis and practical considerations. Diabetes & Metabolic Syndrome: Clinical Research & Reviews. 2020;14(4):303-10.

4.	Yazdani A, Dorri S, Atashi A, Shirafkan H, Zabolinezhad H. Bone metastasis prognostic factors in breast cancer. Breast cancer: basic and clinical research. 2019;13:1178223419830978.

5.	Rondeau V. Statistical models for recurrent events and death: Application to cancer events. Mathematical and Computer modelling. 2010;52(7-8):949-55.

6.	Liu L, Huang X, Yaroshinsky A, Cormier JN. Joint frailty models for zero-inflated recurrent events in the presence of a terminal event. Biometrics. 2016;72(1):204-14.

7.	Jung TH, Kyriakides T, Holodniy M, Esserman D, Peduzzi P. A joint frailty model provides for risk stratification of human immunodeficiency virus-infected patients based on unobserved heterogeneity. Journal of clinical epidemiology. 2018;98:16-23.

8.	Rondeau V, Mathoulin-Pelissier S, Jacqmin-Gadda H, Brouste V, Soubeyran P. Joint frailty models for recurring events and death using maximum penalized likelihood estimation: application on cancer events. Biostatistics. 2007;8(4):708-21.

9.	Jung TH, Peduzzi P, Allore H, Kyriakides TC, Esserman D. A joint model for recurrent events and a semi-competing risk in the presence of multi-level clustering. Statistical methods in medical research. 2019;28(10-11):2897-911.

10.	Fancher KG, Williams MV. Hospital Readmissions: New Pandemic, Same Old Problems? Joint Commission journal on quality and patient safety. 2021;47(11):684-5.

11.	Organization WH. Criteria for releasing COVID-19 patients from isolation: scientific brief, 17 June 2020. World Health Organization; 2020.

12.	Carr E, Bendayan R, Bean D, Stammers M, Wang W, Zhang H, et al. Evaluation and improvement of the National Early Warning Score (NEWS2) for COVID-19: a multi-hospital study. BMC medicine. 2021;19(1):1-16.

13.	Bonanad C, García-Blas S, Tarazona-Santabalbina F, Sanchis J, Bertomeu-González V, Facila L, et al. The effect of age on mortality in patients with COVID-19: a meta-analysis with 611,583 subjects. Journal of the American Medical Directors Association. 2020;21(7):915-8.

14.	Starke KR, Reissig D, Petereit-Haack G, Schmauder S, Nienhaus A, Seidler A. The isolated effect of age on the risk of COVID-19 severe outcomes: a systematic review with meta-analysis. BMJ global health. 2021;6(12):e006434.

15.	Robinson R, Hudali T. The HOSPITAL score and LACE index as predictors of 30 day readmission in a retrospective study at a university-affiliated community hospital. PeerJ. 2017;5:e3137.

16.	Moslemifard M. Hospital diet for COVID-19, an acute respiratory infectious disease: An evidence-based Protocol of a Clinical Trial. Caspian Journal of Internal Medicine. 2020;11(Suppl 1):466.

17.	Zhang L, Zhu F, Xie L, Wang C, Wang J, Chen R, et al. Clinical characteristics of COVID-19-infected cancer patients: a retrospective case study in three hospitals within Wuhan, China. Annals of oncology. 2020;31(7):894-901.

18.	Yang L, Chai P, Yu J, Fan X. Effects of cancer on patients with COVID-19: a systematic review and meta-analysis of 63,019 participants. Cancer Biology & Medicine. 2021;18(1):298.

19.	Abdollahi I, Nabahati M, Javanian M, Shirafkan H, Mehraeen R. Can initial chest CT scan predict status and clinical outcomes of COVID-19 infection? A retrospective cohort study. Egyptian Journal of Radiology and Nuclear Medicine. 2021;52(1):1-10.

20.	Signes-Costa J, Núñez-Gil IJ, Soriano JB, Arroyo-Espliguero R, Eid CM, Romero R, et al. Prevalence and 30-day mortality in hospitalized patients with Covid-19 and prior lung diseases. Archivos de bronconeumologia. 2021;57:13-20.

21.	Rogado J, Pangua C, Serrano-Montero G, Obispo B, Marino AM, Pérez-Pérez M, et al. Covid-19 and lung cancer: A greater fatality rate? Lung cancer. 2020;146:19-22.

22.	Kim S-Y, Yoo D-M, Min C-Y, Choi H-G. The effects of previous hypothyroidism disease on the susceptibility to, morbidity of, and mortality due to COVID-19: A Nationwide Cohort Study in South Korea. Journal of clinical medicine. 2021;10(16):3522.

23.	Du Y, Zhou N, Zha W, Lv Y. Hypertension is a clinically important risk factor for critical illness and mortality in COVID-19: A meta-analysis. Nutrition, Metabolism and Cardiovascular Diseases. 2021;31(3):745-55.

24.	Mazroui Y, Mathoulin‐Pélissier S, MacGrogan G, Brouste V, Rondeau V. Multivariate frailty models for two types of recurrent events with a dependent terminal event: application to breast cancer data. Biometrical Journal. 2013;55(6):866-84.

25.	Mazroui Y, Mauguen A, Mathoulin-Pélissier S, MacGrogan G, Brouste V, Rondeau V. Time-varying coefficients in a multivariate frailty model: Application to breast cancer recurrences of several types and death. Lifetime data analysis. 2016;22(2):191-215.

26.	Li G, Wu Z, editors. An Introduction to Frailty Models for Multivariate Survival Data With Application to Martial Dissolution and Retirement. PAA 2018 Annual Meeting; 2018: PAA.
