# Supplementary material for: Comorbidities affecting re-admission and survival in COVID-19: Application of joint frailty model
Source: PLoS One. 2024 Apr 18;19(4):e0301209. doi: 10.1371/journal.pone.0301209 (PMC11025956; doi:10.1371/journal.pone.0301209)
Supplement: Attachment — Submitted filename: PLOS One review for.docx [file pone.0301209.s004.docx]

PLOS One review for

**Title:** *Comorbidities affecting re-admission and survival in COVID-19: Application of joint frailty model*

**Comments to the authors:**

I commend the authors for exploring the important topic of readmission after the COVID-19 disease. The authors performed a comprehensive collection of the available data. Using the joint frailty model is a strength of this study.

**Abstract:**

The abstract needs to be structured more fluently and use consistent terminology (e.g., “re-admission” versus “readmission”). Some numbers need to be spelled out (more than twice versus “ more than 2 times”). I suggest introducing the hazard of death influence in the order of strength: hyperlipidemia (HR 4.05), followed by age (HR 1.76), and cancer (1.64).

**Introduction:**

This part is improved following the suggestions and comments from “A.” However, some inconsistencies regarding terminology and grammar errors exist.

**Material and Methods:**

**Study design:**

The inclusion criteria for this study are marked by bias. Some patients could have fallen out of the study observation without being admitted to the same hospital system since the follow-up ended with the end of the study (July 2021).

**Study variables:**

Using the NEWS2 score to assess the severity of COVID-19 can be debatable since the authors of the article that introduced the score mentioned poor-to-moderate discrimination for medium-term COVID-19 outcomes.

**Results:**

The data is presented in a complicated, overfilled-with-numbers manner that is difficult to follow. Again, I suggest consistency in terminology; there are too many terms of the same meaning ("re-admission," "readmission," "re-hospitalization").

**Discussion:**

The authors should give the most plausible explanation as to why the history of lung diseases was protective. I also recommend being consistent and using either lower or capital letters for diseases such as malignancy.
