# Supplementary material for: Comorbidities affecting re-admission and survival in COVID-19: Application of joint frailty model
Source: PLoS One. 2024 Apr 18;19(4):e0301209. doi: 10.1371/journal.pone.0301209 (PMC11025956; doi:10.1371/journal.pone.0301209)
Supplement: Attachment — Submitted filename: October Review PLOS One for COVID-19 paper.docx [file pone.0301209.s006.docx]

PLOS One review for PONE-D-23-04746R1

**Title:** *Comorbidities affecting re-admission and survival in COVID-19: Application of joint frailty model*

**Comments to the authors:**

Again, I commend the authors for exploring the important topic of readmission after the COVID-19 disease. The authors performed a comprehensive collection of the available data. Using the joint frailty model is a strength of this study. The authors addressed many of the reviewers’ suggestions. Unfortunately, the clarity and fluidity in English continue to be a problem overall, and I recommend using an editor who can proofread and correct the English semantics and grammar.

**Abstract:**

The authors incorporated the suggestions for the abstract. The content reads more clearly and concisely.

**Introduction:**

Please correct linguistic inconsistencies and grammar errors. Consider using a native English speaker editor.

For example the line 58-59 in the Introduction should read “ *Recognizing the factors affecting re-admission, management, and policy planning can help provide appropriate and optimal health care”*  and 70-72 should read “*In COVID-19 infection, patients may be re-hospitalized after initial treatment and discharge with different causes (including respiratory distress, metabolic encephalopathy, etc.)”*

**Material and Methods:**

**Study design:**

Some patients could have fallen out of the study observation without being admitted to the same hospital system since the follow-up ended with the end of the study (July 2021). The authors addressed this in the discussion as a study limitation.

**Study variables:**

Using the NEWS2 score to assess the severity of COVID-19 can be debatable since the authors of the article that introduced the score mentioned poor-to-moderate discrimination for medium-term COVID-19 outcomes. This was addressed in one sentence in the Methods (line 132-133).

**Results:**

The data continues to be presented in a complicated, overfilled-with-numbers manner that is difficult to follow. I suggest using the same pattern they used in the abstract: for instance in the Abstract the authors used “*mean (SD) age of 63.76 (14.58)” versus” mean (±SD) age of 63.76 (±14.58)” in the results.*

**Discussion:**

The Discussion part should be more elaborated, not only with insertions from medical literature but to address possible explanations for their study results. There are significant linguistic inaccuracies that need to be corrected as well.

**Confidential to the Editor:**

**The authors The information flow continues needs significant improvement for coherence and clarity. I recommend a statistician review of the Results. I suggest the authors perform a major revision before publication.**
