# Supplementary material for: Comorbidities affecting re-admission and survival in COVID-19: Application of joint frailty model
Source: PLoS One. 2024 Apr 18;19(4):e0301209. doi: 10.1371/journal.pone.0301209 (PMC11025956; doi:10.1371/journal.pone.0301209)
Supplement: Attachment — Submitted filename: December review for PLOS One COVID manuscript.docx [file pone.0301209.s007.docx]

PLOS One review for PONE-D-23-04746R1

**Title:** *Comorbidities affecting re-admission and survival in COVID-19: Application of joint frailty model*

**Comments to the authors:**

Overall, the authors addressed most of the suggestions. Although this is a small study, I commend the authors for evaluating this important topic of readmission after COVID-19 infection and its association with mortality.

I recommend that the authors add their explanation of why the risk of death increased with a history of cancer and hyperlipidemia. The discussion concentrated more on the cancer history and failed to elaborate on hyperlipidemia as a risk factor.
