# Supplementary material for: Comorbidities affecting re-admission and survival in COVID-19: Application of joint frailty model
Source: PLoS One. 2024 Apr 18;19(4):e0301209. doi: 10.1371/journal.pone.0301209 (PMC11025956; doi:10.1371/journal.pone.0301209)
Supplement: Attachment — Submitted filename: review.docx [file pone.0301209.s008.docx]

Thank you for the opportunity to review this manuscript. Many reviews have been done on this article, which has made it better than its initial version.

this is a good study on the joint frailty model.

Some minor points to be considered and fixed before publication:

What do you mean confirming of covid-19 by imaging or laboratory documents In line of 116? CT scan and what laboratory finding ?

Why is NEWS2 finding not in the table? explanation in Line 190-193

It seems that the number of survivors In table 1 is wrong and should be corrected 64.
