# Supplementary material for: Comorbidities affecting re-admission and survival in COVID-19: Application of joint frailty model
Source: PLoS One. 2024 Apr 18;19(4):e0301209. doi: 10.1371/journal.pone.0301209 (PMC11025956; doi:10.1371/journal.pone.0301209)
Supplement: Attachment — Submitted filename: response.docx [file pone.0301209.s009.docx]

**Dear Editor of PLOS ONE**

I would like to submit our revision based on the comments of reviewers in the research article: ***“*Comorbidities affecting re-admission and survival in COVID-19: application of joint frailty model*”*** with the code: PONE-D-23-04746R1***.***

We tried to correct the manuscript based on the comments of reviewers point by point.

Sincerely yours

Hoda Shirafkan, PhD

Assistant Professor in Biostatistics;

Department of Community Medicine;

School of Medicine;

Babol University of Medical Sciences, Babol, Iran.

Tel: +98 9112181439

| comment | answer |
| --- | --- |
| What do you mean confirming of covid-19 by imaging or laboratory documents In line of 116? CT scan and what laboratory finding? | Confirmation of COVID-19 was done by RT-PCR test (a laboratory document) or by imaging or laboratory documents like CRP) |
| Why is NEWS2 finding not in the table? explanation in Line 190-193 | Should all the findings be mentioned again in the table? Considering that the severity of the disease was different in each hospitalization, we only evaluated the severity of the disease in the first visit and reported it in the text of the article. |
| It seems that the number of survivors In table 1 is wrong and should be corrected 64 | We corrected it. |

Email: hodashirafkan@gmail.com
